# Diabetes self-management arrangements in Europe: a realist review to facilitate a project implemented in six countries

**DOI:** 10.1186/1472-6963-14-453

**Published:** 2014-10-02

**Authors:** Antonis A Kousoulis, Evridiki Patelarou, Sue Shea, Christina Foss, Ingrid A Ruud Knutsen, Elka Todorova, Poli Roukova, Mari Carmen Portillo, María J Pumar-Méndez, Agurtzane Mujika, Anne Rogers, Ivaylo Vassilev, Manuel Serrano-Gil, Christos Lionis

**Affiliations:** Clinic of Social and Family Medicine, Faculty of Medicine, University of Crete, Voutes, P.O. Box 2208, Heraklion 71003 Greece; Florence Nightingale School of Nursing and Midwifery, King’s College London, London, UK; Institute of Health and Society, University of Oslo, Oslo, Norway; Department of Economic Sociology, University of National and World Economy, Sofia, Bulgaria; NIGGG, Bulgarian Academy of Sciences, Sofia, Bulgaria; School of Nursing, University of Navarra, Pamplona, Spain; NIHR CLAHRC Wessex, Health Sciences, University of Southampton, Hampshire, UK; Fundación Educación Salud y Sociedad, Murcia, Spain

**Keywords:** Chronic disease, Diabetes mellitus, Europe, Government, Delivery of health care, Health policy, Health personnel, Self-care, Social welfare

## Abstract

**Background:**

Self-management of long term conditions can promote quality of life whilst delivering benefits to the financing of health care systems. However, rarely are the meso-level influences, likely to be of direct relevance to these desired outcomes, systematically explored. No specific international guidelines exist suggesting the features of the most appropriate structure and organisation of health care systems within which to situate self-management approaches and practices. This review aimed to identify the quantitative literature with regard to diabetes self-management arrangements currently in place within the health care systems of six countries (The United Kingdom, The Netherlands, Norway, Spain, Bulgaria, and Greece) and explore how these are integrated into the broader health care and welfare systems in each country.

**Methods:**

The methodology for a realist review was followed. Publications of interest dating from 2000 to 2013 were identified through appropriate MeSH terms by a systematic search in six bibliographic databases. A search diary was maintained and the studies were assessed for their quality and risk of bias.

**Results:**

Following the multi-step search strategy, 56 studies were included in the final review (the majority from the UK) reporting design methods and findings on 21 interventions and programmes for diabetes and chronic disease self-management. Most (11/21, 52%) of the interventions were designed to fit within the context of primary care. The majority (11/21, 52%) highlighted behavioural change as an important goal. Finally, some (5/21, 24%) referred explicitly to Internet-based tools.

**Conclusions:**

This review is based on results which are derived from a total of at least 5,500 individuals residing in the six participating countries. It indicates a policy shift towards patient-centred self-management of diabetes in a primary care context. The professional role of diabetes specialist nurses, the need for multidisciplinary approaches and a focus on patient education emerge as fundamental principles in the design of relevant programmes. Socio-economic circumstances are relevant to the capacity to self-manage and suggest that any gains and progress will be hard to maintain during economic austerity. This realist review should be interpreted within the wider context of a whole systems approach regarding self-care support and chronic illness management.

**Electronic supplementary material:**

The online version of this article (doi:10.1186/1472-6963-14-453) contains supplementary material, which is available to authorized users.

## Background

According to the World Health Organization, long term conditions are illnesses of long duration and generally slow progression; they are by far the leading cause of mortality in the world [1]. Insufficiently controlled chronic conditions can lead to more rapid deterioration, complications, poor quality of life and increased use of the health care services [2]. Within this context, effective disease self-management – according to new models of chronic care [3] – can help to empower the patient and may aid in promoting a person’s dignity, self-determination, and well-being, while at the same time, potentially achieving operational savings within health care systems through a reduction in utilisation. The latter is particularly important during a period of economic crisis where health care systems and welfare regimes are affected [4]. Currently, self-management support forms a central aspect of chronic illness management nationally and globally, although evidence of its success has mainly focused on individually-centred outcomes of behavioural change [5].

As Europe is facing unprecedented social, economic, demographic and epidemiologic transitions, the discussion on the structure and governance of health care systems is of primary importance as major influences on self-management support infrastructures and processes. Long term conditions are the primary focus of interest, accounting for more than 30 million deaths per year and heavy health care utilisation [1]. Even though these are a central cause of quality of life deterioration, carefully designed self-management programmes are not systematically implemented to release some of this burden.

Public policy and the organisation of health and welfare shape to a large extent how policies such as self-management are enacted in practice but are rarely taken into consideration in understanding the potential in terms of health and social outcomes [6]. Initiatives towards an enhanced implementation of diabetes self-management have been variably directed by various actors. Government policy, Non-Governmental-Organisations (NGOs), relevant professional groups, and health and non-health care sector providers, political will, decisions by regional boards responsible for providing or commissioning services, and private input all have impact as part of such initiatives and thus emerge as relevant issues of interest [7–9] .

### The EU-WISE Project

The EU-WISE collaborative project (http:http://www.eu-wise.com) involves 6 countries including The United Kingdom, The Netherlands, Norway, Spain, Bulgaria, and Greece with the aim to produce a theoretical and methodological background of systems of support, summarise the structure and governance of health and welfare systems of the six participating countries and to describe, explain and profile the structure of personal networks of people with diabetes in different national contexts. Moreover, this project aims to describe and analyse the support mechanisms that operate on the meso-level, to survey the systems of support of people with diabetes and the networks of the organizations. Finally, the EU-WISE collaborative project aims to identify existing mechanisms involved in diabetes’ management with the aim to develop and implement new strategies of engagement and support self-care.

### Aim and research questions

The realist review reported here will inform the second work package of this EU project through the identification of quantitative published peer-reviewed literature with regard to diabetes self-management arrangements currently in place (within the health care systems of the different partner countries) and how these are integrated into the broader welfare systems. Within the EUWISE framework, it was agreed to explore the existing different welfare policies, systems provision, structure and reforms of partner healthcare systems and association through direct and indirect impact on the configuration of support for people with chronic illness, with a focus on those with type 2 diabetes.

This realist review aims to assess the key government objectives and direction of change in the provision of self-care and how the role of health care professionals is changing within this environment. Whilst for the purpose of this online search a number of research questions were identified, and they were in line with those raised during the formation of the research protocol, as the methodology of the realist review suggests, the purposes of the review were refined during the process of the literature search. Eventually (and primarily after the background search that gave a feel for the literature), the key themes to explore were guided by the following questions:

1. What is the content of policies directed at diabetes’ self- management support over the last 10-15 years in the 6 participating countries (The United Kingdom, The Netherlands, Norway, Spain, Bulgaria, and Greece)?
2. Are there any governmental or other initiatives, arrangements, actions or interventions towards self-care adoption and behavioural change for chronic disease management in general and diabetes in particular?
3. To what extent do the current government actions are structured towards the provision of health care through behaviour change and self-care?
4. What changes in usual care and resources have been noticed during the last 10-15 years in the six participating countries, given the impact of the changing socioeconomic conditions?
5. How have the roles of health care professionals changed in regards to diabetes management and in what ways have the configurations of the professional-patient relationships in services to influence self-management changed over the last 10-15 years?

## Methods

### Realist review and search strategy

A realist review is a systematic method which has been used for assessing complex interventions, focused on identifying determinants of their impact, and evaluating or developing theories [10]. The aim of the realist review is to develop concepts and knowledge about variations in a phenomenon rather than seeking one comprehensive effect size. Furthermore, it can be used with quantitative, qualitative and multiple methods [11].

We drew on the principles of the realist review as this strategy for synthesising research aims to unpack the mechanisms of how complex programmes work in particular contexts and settings (what works for whom and in what circumstances). As first step in the design of the review, the underlying assumptions about how an intervention is meant to work and what impacts it is expected to have were identified and the relevant theoretical framework was put together [11]. To that end, a literature review to summarise the peer-reviewed published scientific literature in the field (stage 1) was followed. Relevant papers were then synthesised narratively (stage 2).

Stage 1 included identification of all material which had been published following a peer-reviewed process (original or policy articles). A search diary was maintained detailing the names of the databases that were searched, the keywords used and the search results.

For the literature search, a systematic approach was undertaken, utilising the specific keywords and criteria, as indicated below. The methodology of the realist review, that combines theoretical understanding with empirical evidence, was followed throughout [11]. The studies identified were grouped and presented in summary tables featuring the key messages of each one.

### Information resources – Search terms

Publications of interest were identified by a search of the following information resources since 2000: PubMed, Scopus, Web of Science, PsycINFO, European Observatory on Health Systems and Policies, Centre for Reviews and Dissemination - Database of Abstracts and Reviews of Effects (DARE).

**Figure 1:**
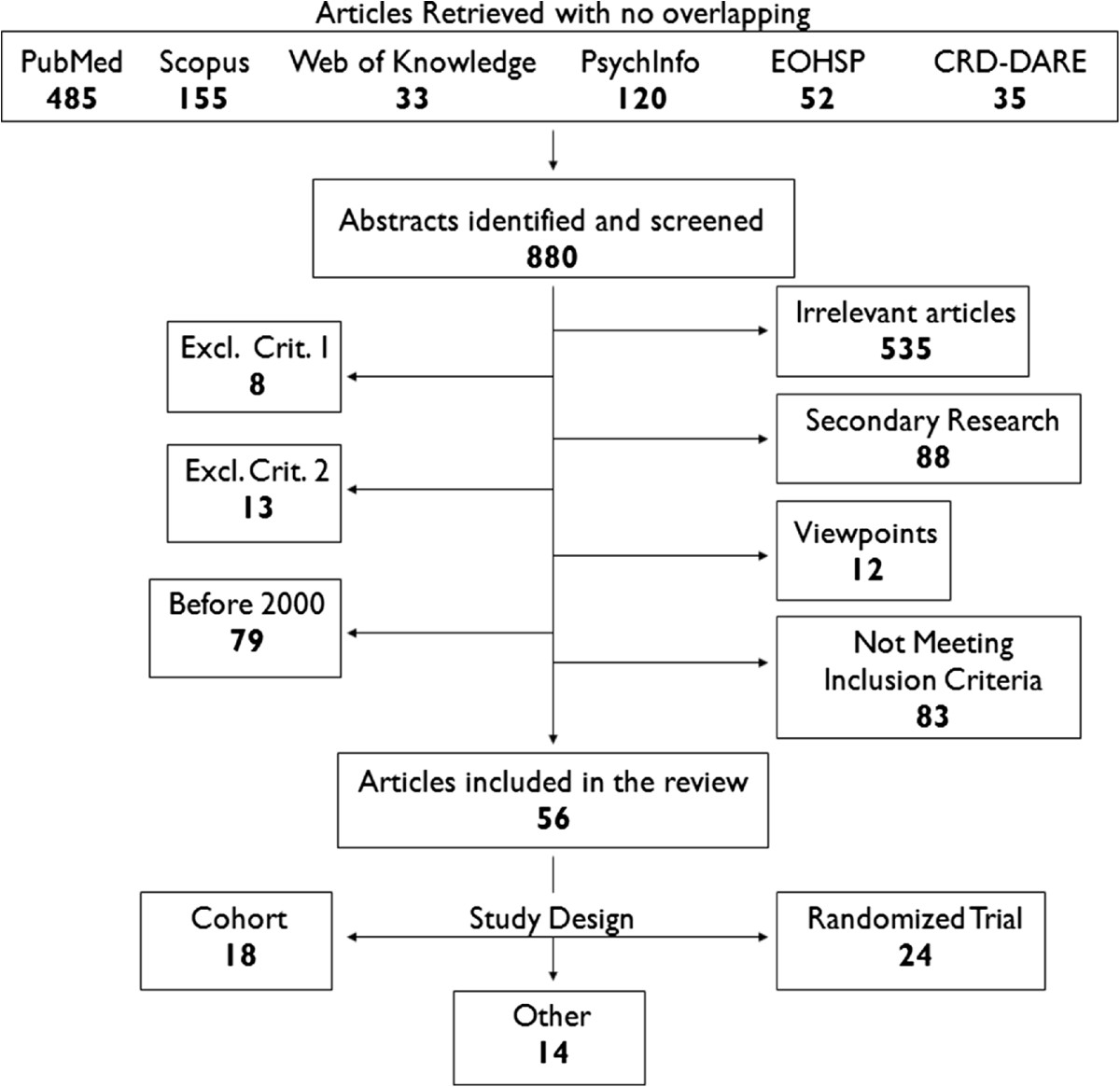
**Flowchart for the literature review.**

Various search terms were used to include both MeSH terms and other glossary databases. Guided by entry terms in MeSH and mapped terms in Emtree thesaurus, the main ones used were: Chronic Disease, Diabetes Mellitus, Self Care Support, Self Care Management, Health Care System, Social Welfare, Chronic Illness Management, Chronic Illness Policy, Self Management Systems, Health Resources, Health Care Providers, Health Plan Implementation, Government Programmes, Skill-Mix. Within this context, indicatively, the electronic search strategy that was followed was constructed on the following search algorithm for Medline bibliographical database: *(diabetes [Title/Abstract]) AND (self care support OR self care management) AND (health care system OR social welfare OR policy OR providers OR government OR skill-mix)*. Advanced search options have been used to combine the terms (and their truncations) in every database.Thereafter, reference lists were systematically searched for further relevant articles by two reviewers; finally, corresponding authors were contacted with regard to missing data. A relevant flow chart was constructed to detail the number of papers retrieved and the steps undertaken (Figure 1).

### Eligibility criteria

The specific inclusion and exclusion criteria that have been proposed in the review protocol are illustrated in Table 1.

**Table 1:** **Specific inclusion and exclusion criteria**

Inclusion	Exclusion
1. Published articles providing information and evidence relevant to the management of diabetes mellitus in health care services and its integration in the broader welfare systems.	1. Evidence published in languages other than English (except each partner specific language).
2. Published articles reporting government initiatives, actions, interventions and specific country policies that promote self-care adoption and behaviour change interventions in patients with chronic illness.	2. Published articles reporting government initiatives, actions, interventions and specific country policies that promote self-care adoption and behaviour change interventions in patients without chronic illness.
3. Published articles reporting methods and tools used in interventions that promote self-care adoption and behaviour change in patients with chronic illness.	
4. Published articles reporting and discussing the role of key professional groups and particularly health care professionals in interventions that promote self-care adoption and behaviour change in patients with chronic illness.	

### Data collection and management process

A standard ‘search diary’ was maintained detailing the names of the databases that were searched, the keywords used and the search results. Titles and abstracts of studies to be considered for retrieval were recorded on a form, along with details of where the reference had been found. Inclusion and exclusion decisions were recorded on a database. Retrieved studies were filed according to inclusion and exclusion decisions. Studies were selected for retrieval after two independent reviewers had appraised titles and abstracts identified in electronic searches. All references provided by expert contacts were also retrieved.

Two reviewers (AK, EP) independently abstracted data and all relevant information, which was placed onto a form and summarised in order to identify what was considered to be the most important results from each study. These summaries were compared and any differences of opinion were resolved by discussion and consultation with the original study.

### Data synthesis and risk of bias in individual studies

For the narrative synthesis, the studies were grouped and presented in Summary Tables featuring key points of each study (Tables 2 and 3). A standard form was used to assist the sorting of primary source material, but in the context of the realist review a bespoke form was developed to serve a different purpose (see Table 3). Preliminary primary sources (as in the paper by Bower et al. for example [12]) were included in the results as it is important in a realist review to identify possible relevant concepts and theories [11]. Furthermore, in terms of quality appraisal and to identify fatally flawed empirical papers (which in a realist review differs from the methodological approach of a systematic review), we decided to assess the five prompts suggested by Dixon-Woods et al for all studies: (i) Are the aims and objectives of the research clearly stated? (ii) Is the research design clearly specified and appropriate for the aims and objectives of the research? (iii) Do the researchers provide a clear account of the process by which their findings were reproduced? (iv) Do the researchers display enough data to support their interpretations and conclusions? (v) Is the method of analysis appropriate and adequately explicated? [13]. The quality appraisal of the evidence occurred in parallel with the synthesis and narration of results, and judgment had to be used to supplement the formal critical appraisal checklist and consider all included articles in terms of relevance and rigour [11].

**Table 2:** **An overview of the studies included in the review**

Country	Initiative	Relevant citations	Definition	Goals to be obtained	Overall studies participants	Main research findings	Setting	Professionals roles
UK	Local Diabetes Centres (within The Diabetic Retinopathy Screening Service for Wales)	Dennis et al, 2000[14]	Education, Community based service, Real and virtual specialist support service, Enhanced all-Wales screening service.	Behaviour change.	Locally implemented	Some elements (funding, structure have been successful in promoting self-management, but some need revising (education, behavioural change). There are 3 models of diabetes education in Wales. More to be invested in this area in the years to come.	Primary Care	Patient education a requirement of all providing services to patients with diabetes. Staff encouraged to set an example.
UK	DAFNE (Dose Adjustment For Normal Eating)	Jack, 2001; DAFNE Study Group, 2002; Shearer et al, 2004; Speight et al, 2010; Lawton et al, 2010; Rankin et al, 2011; Leelarathna et al, 2011; Keen et al, 2012; Gunn et al, 2012; Rankin et al, 2012 [15–24]	A course teaching flexible intensive insulin treatment combining dietary freedom and insulin adjustment (delivered in 35 hours over 5 consecutive days).	Dietary freedom.	715	Improved quality of life (p < 0.001) and glycaemic control (p < 0.0001) in people with type 1 diabetes without worsening severe hypoglycaemia or cardiovascular risk. Has the potential to be a cost-saving initiative. The impact of a single DAFNE course on glycaemic control remains apparent in the long term (4 years). DAFNE delivered in routine clinical practice is associated with a range of benefits and certain clinical and psychosocial characteristics are associated with better outcomes. Results show significant reductions in total, quick acting and basal insulin (all p < 0.0005) doses in patients undergoing DAFNE training.	Secondary Care - Diabetes Clinics	Diabetes specialist nurses and dieticians who attended a training course.
UK	LAY (Look After Yourself programme)	Cooper et al, 2003a; Cooper et al, 2003b; Cooper et al, 2008 [25–27]	Theoretically constructed on the premise that knowledge acquisition alone does not necessarily promote self-directed action. Rather, systems of motivation and the teaching of skills (practical, physical, conceptual, emotional, social and personal) are stressed.	Behaviour change, empowerment-based education.	89	Associated with only limited benefits in glycaemic control (only significant in 6 months, p < 0.005), but there were significant educational (p < 0.002) and psychological benefits.	Primary Care/ Hospital	Diabetes specialist nurses trained in the programme.
UK	Portsmouth Primary Care Trust, Self-management programmes for people with diabetes	Cradock, 2004 [28]	Structured self management programmes, delivered to groups of patients, to assist in helping people be clearer about how they can make changes that will reduce their risk of diabetes complications and cardiovascular disease.	Behaviour change.	Locally implemented	Engaging with patients in a group situation appears to be beneficial. The programme has run since 2001 and the evidence is that it is working (3 practices added group follow-up).	Primary Care	Nurses and dieticians. Training around empowerment, counselling and communication skills.
UK	UCL-DSMP (University College London-Diabetes Self Management Programme)	Steed et al, 2005 [29]	Group-based programme consisting of five 2.5 hour sessions held weekly for five weeks, plus one booster session of 2.5 hours held three months after the end.	Behaviour change, quality of life.	124	At immediate post-intervention and three-month follow-up the intervention group showed significant improvement relative to controls on self-management behaviours (p < 0.01), quality of life (p < 0.01) and illness beliefs (p < 0.05). A trend towards improved HbA1c was also observed (p < 0.01).	Outpatient clinics, hospital	Diabetes specialist nurses and dieticians.
UK	Librae	Franklin et al, 2006 [30]	Software package in the form of ‘diabetes diary’ (validated algorithm) to input data related to patients’ daily diabetes self-management.	Individual responsibility, Educational predictive tool.	15	The modelled values of ‘Librae’ correlated well with the continuous blood glucose monitoring data (positive mean 0.35 mmol/L), but clinically unacceptable errors occurred at extremes of blood glucose levels.	Diabetes Clinic	No direct health care professional input.
UK	Diabetes Manual	Sturt, Hearnshaw et al, 2006; Sturt, Taylor et al, 2006; Sturt et al, 2008; Lindenmeyer et al, 2010 [31–34]	A self-management 1:1 educational intervention aimed at improving biomedical and psychosocial outcomes.	Behavioural change, skills and confidence for self-management.	257	A small improvement in patient diabetes-related distress (p = 0.012) and confidence to self-care over 26 weeks, but no significant difference in HbA1c (p = 0.39). The programme requires close communication and openness towards collaborative approaches to improve skills and confidence for self-management.	Primary Care	2-day training for nurse to deliver the programme; telephone-support in weeks 1,5,11; 12-month follow-up.
UK	DESMOND (Diabetes Education and Self-Management for Ongoing and Newly Diagnosed)	Skinner et al, 2006; Davies et al, 2008; Ockleford et al, 2008; Skinner et al, 2008; Gillett et al, 2010; Skinner et al, 2011; Khunti et al, 2012 [35–41]	Structured education program on illness beliefs, quality of life and physical activity.	Behavioural change, illness awareness, lifestyle outcomes.	1660	Newly diagnosed individuals are open to attending self-management programs. Positive improvements in beliefs about illness and weight loss. Structured group education is essential. Combining illness beliefs into discrete clusters may be more useful in understanding patterns of responding to illness. The intervention is likely to be cost effective compared with usual care. A single programme for people with newly diagnosed type 2 diabetes showed no difference in HbA1c (P = 0.81) or lifestyle outcomes at 3 years, but illness belief score differed significantly (p = 0.01).	Primary Care	Specific guidelines for trained educators. The amount of time educators talk provides practical marker for the effectiveness of the process.
UK	The diabetes X-PERT programme	Deakin et al, 2006; Deakin et al, 2009; Choudhury et al, 2009 [42–44]	6-week structured education programme based on theories of patient empowerment and discovery learning, to develop skills and confidence leading to increasing diabetes self-management and sustain improvement.	Personal responsibility, lifestyle and psychosocial outcomes.	191	Attendance rates 58%. Participation in the X-PERT Programme by adults with T-2D was shown at 14 months to lead to improved glycaemic control, reduced total cholesterol level, body weight, BMI & waist circumference, reduced requirement for diabetes medication, increased consumption of fruit and vegetables, enjoyment of food, knowledge of diabetes, self-empowerment, self-management skills and treatment satisfaction (all self-reported).	Primary Care	The programme trains health-care professionals to deliver it to people with diabetes.
UK	BITES (Brief Intervention in Type 1 diabetes, Education for Self-efficacy)	George et al, 2007; George et al, 2008 [45, 46]	Brief (2.5 days) psycho-educational intervention	Patient empowerment	114	At 12 months, had no significant impact on HbA1c (p = 0.94) or severe hypoglycaemia, but improved diabetes treatment satisfaction (p = 0.006) and patient empowerment.	Secondary Care	Nurses and dieticians. Multidisciplinary teams.
UK	Diabetes Virtual Clinic	Armstrong et al, 2008; Jennings et al, 2009; Powell et al, 2009; Armstrong et al, 2012 [47–50]	Internet-based self-management tool for diabetes allowing patients to communicate with their health professionals, find information about their condition and share support and advice with others through peer-to-peer discussions.	User-centred approach, Support for patients to become effective self-managers	22	The pilot study did not identify evidence of an impact on HbA1c (p = 0.53), improving quality of life or self-efficacy in patients who used insulin pump therapy. Users found participation reassuring. They rated peer interaction (53%) as the most desirable and the most useful of the features available.	Hospital clinics (online community)	Online “ask an expert” sessions conducted with diabetes specialists not directly involved with the patients care.
UK	Birmingham Own Health telephone care management service	Jordan et al, 2011 [2]	Telephone-based care service (nurse-delivered motivational coaching and support for self-management and lifestyle change) for patients with poorly controlled diabetes.	Behavioural, lifestyle change.	473	The intervention is effective in reducing HbA1c levels (p = 0.0004), blood pressure and BMI in people with diabetes. Study design had limitations (controls matched from a retrospective cohort).	Primary Care (telephone-based)	Specifically trained nurses as Care Managers.
UK	Whole Systems Model	Bower et al, 2012 [12]	Self-management support through an evidence-based ‘whole systems’ model involving patient support, training for primary care teams, and service re-organisation, all integrated into routine delivery within primary care.	Behaviour change, Whole System Approach	Designed	*Protocol paper only*	Primary Care	Multidisciplinary approach
Netherlands	CBGT (Cognitive Behavioural Group Training)	Snoek et al, 2001; van der Ven et al, 2005a; van der Ven et al, 2005b [51–53]	4 weeks cognitive behavioural small group training aimed at modifying dysfunctional beliefs, reducing negative emotions and enhancing self-care practices.	Behavioural change	131	Following CBGT, mean HbA1c dropped by 0.8% at 6 months from baseline (p = 0.36), while emotional well-being was preserved. CBGT was successful in improving self-efficacy (p = 0.01), diabetes-related distress (p = 0.01) and mood (p < 0.001) at 3 months’ follow-up, but not in improving glycaemic control.	Outpatient setting	Diabetes nurse specialist and psychologist.
Netherlands	Theory-driven Intervention	Schreurs et al, 2003 [54]	Action plans to enhance self-management provided to disease-homogeneous groups of patients.	Planning of behaviour, goal-setting	24	The majority of participants were satisfied with the programme and positive about most of the intervention aspects (evaluation scores ranged 3.03-4.05/5). Patients of older age, lower education, or no current employment responded best to the intervention.	Outpatient department, hospital	Specialised nurses trained by cognitive behavioural therapists, techniques applicable in the daily care.
Netherlands	Di@alog Study	Roek et al, 2009 [55]	Web-based self-management programme for insulin titration in T2DM patients.	Personal responsibility, glycaemic control	Designed, 248	Protocol paper only	Primary Care (web based)	General Practitioner and practice nurse more conscious of the treatment process.
Netherlands	Diabetes Coach	Nijland et al, 2011 [56]	Web-based application for supporting the self-care of patients with type 2 diabetes.	Empowered patients	50	Long diabetes duration a factor for increased engagement (p = 0.03). Factors influencing increased use of eHealth technologies: (1) avoiding selective enrollment, (2) making use of participatory design methods, and (3) developing push factors for persistence.	Primary Care (web based)	Multidisciplinary teams, patient-nurse email exchange.
Netherlands	DIEP.info (Diabetes Interactive Education Programme)	Heinrich et al, 2012 [57]	Web-based type 2 diabetes self-management education programme aimed at improving knowledge, encouraging active patient participation and providing supportive self-management tools.	Knowledge improvement	674	The effect evaluation showed a significant intervention effect (p < 0.01) on knowledge. The user evaluation showed high satisfaction with the programme’s content, credibility and user-friendliness. However, it is not fully used as intended.	Web-based	Active role and clear instructions for health care professionals.
Norway	Diabetes Self Management Education	Rygg et al, 2010; Rygg et al, 2012 [58, 59]	Locally developed group based education.	Knowledge improvement, skills	168	The controls in locally developed ongoing diabetes self-management education programs prevented an increase (0.3%) in HbA1c and can have an effect in patients with higher levels. Locally developed education programmes seem to have less effect than interventions developed for studies.	Hospital	Led by diabetes nurses, and input by physician, physiotherapist and a lay person.
Spain	eHealth platform	Fico et al, 2011; Fioravanti et al, 2011 [60, 61]	Technological platform for diabetes disease management.	Web usability to induce self-care	23	High usability and satisfaction (score 4.7/6).	Web-based	Clinicians, market analysis and technology experts.
Bulgaria	DEPB (Diabetes Education Program in Bulgaria)	DEBM, 2001[7]	A large-scale unified structured educational programme for insulin-treated diabetic patients.	Education, knowledge improvement, empowered patients	1037	56 educational centres. Trained patients cope better with their condition.	Regional centers (potential for primary care)	Endocrinologist, nurse.

**Table 3:** **Background on the implementation of the selected initiatives**

Initiative	Country	Background information
Diabetic Retinopathy Screening	UK	Political commitment to an all-Wales screening service [14, 62].
DAFNE	UK	Multicentre trial under Diabetes UK, based on programme developed by a German group [15, 17].
LAY	UK	The philosophy and push came from the Long Term Condition Alliance. Signed up to the US scheme of LTCM [25, 63, 64].
Portsmouth Primary Care Trust	UK	Started outside governmental knowledge on EPP, in collaboration with Portsmouth Hospitals NHS Trust [28].
UCL-DSMP	UK	University-run trial [29].
Librae	UK	Model with the intention to enter routine clinical practice [30].
Diabetes Manual	UK	University-developed package [32, 65].
DESMOND	UK	Collaborative of NHS organisations, co-ordinating centre hosted by University Hospitals of Leicester NHS Trust [41, 66].
X-PERT	UK	Variant on the DESMOND programme, local factors implicated [44].
BITES	UK	Variant on the DAFNE programme, university funded [45].
Diabetes Virtual Clinic	UK	Pilot study, internally university funded [48, 50].
Birmingham Own Health	UK	Sponsored in part by the private sector in a mixed model of health economy [2, 67].
Whole Systems Model	UK	WISE, funded by National Institute for Health Research and National Primary Care Research and Development centre [12].
CBGT	Netherlands	Study funded by pharmaceutical funding [52].
Theory-driven Intervention	Netherlands	Intervention developed locally. Philosophy came from US scheme [54, 64].
Di@log	Netherlands	Trial funded by pharmaceutical funding [55].
Diabetes Coach	Netherlands	Study supported by local primary healthcare foundation and home care organisation [56].
DIEP.info	Netherlands	University-run local programme [57].
Diabetes Self-Management Education	Norway	Supported by state-owned authorities [59].
eHealth	Spain	Partially funded by the European Commission, under the 7th Framework Programme [60].
DEPB	Bulgaria	Implemented by the Ministry of Health of Bulgaria [7].

## Results

### Literature review

In this section the raw data results from the literature search are presented. A more elaborated presentation of the results, in the context of the realist study and its conclusions in terms of factors potentially relating to the variation in the phenomena of interest, is detailed further in the Discussion section.

The multi-step search strategy yielded 880 documents from six bibliographical databases. As a result of the evaluation, 535 articles were deemed irrelevant, 101 matched the exclusion factors, 83 did not fit the inclusion criteria as outlined in Table 1, and 106 did not constitute original research. All in all, 56 studies were included in the final review reporting design methods and findings on 21 interventions and programmes for diabetes and chronic disease self-management, since 2000 (Figure 1). Twenty-four out of the 56 studies were randomised controlled trials, 18 used a cohort design, and 14 followed other designs including cost-effectiveness analyses, complex intervention framework and presentations of programmes. The themes discussed by all were revolving around the same basic axes. Secondary research and relevant systematic reviews were only later integrated to further enhance the Discussion. Thirteen of the studies (61.9%) came from the The United Kingdom, five (23.8%) from The Netherlands, one (4.76%) from Norway, one (4.76%) from Spain, and one (4.76%) from Bulgaria; only initial discussions and approaches have taken place in Greece [68]. Characteristics and detailed information of the included studies are listed in Table 2. Relevant productive information on the background to the introduction of the selected initiatives is included in Table 3.

Initiatives that only provided information in a didactic format or manipulated delivery of information (e.g. assessment of telephone consultations) were not included because provision of information alone has been recognised as insufficient for improved self-management [69]. Nevertheless, information and contextual aspects of these papers have been discussed. Many (11/21, 52%) of the interventions were designed to fit within the context of general practice as the primary setting. Fifty-two per cent (11/21) clearly stated behavioural change as an important goal, whereas the concepts of empowering patients and addressing individual responsibility were stated in the remaining 48% (10/21). Five interventions (24%) referred to Internet-based tools.

The professional role of diabetes specialist nurses, the need for multidisciplinary approaches and the focus on patient education emerge as fundamental principles in the design of relevant programmes from the majority of the papers (16/21, 76%). The results of the current review, indicating a shift towards patient-centred self-care of diabetes, derive from a total of at least 5,500 individuals residing in the six participating countries.

## Discussion

### What was revealed in this review?

As evident by the review, success of self-management as a policy solution will be affected by interacting influences at three levels: [a] at micro-level by individuals’ dispositions and capabilities; [b] at meso-level by roles, relationships and material conditions within the family and in the workplace, school and healthcare organisation; and [c] at macro-level by prevailing economic conditions, cultural norms and expectations, and the underpinning logic of the healthcare system [8]. Even though interventions are usually developed for specific chronic diseases, the themes and behavioural strategies are comparable across various disease-homogeneous groups of patients. All chronically ill patients face psychological demands, such as maintaining an optimal quality of life, preserving a reasonable emotional balance and sustaining relationships with family and friends [54]. Social networks, especially, seem to offer an opportunity to redress the balance of an exclusively individual focus on self-management [70], because they address the broader set of contributions and resources available to people in need of chronic illness management and support [5].

In an overview of its key findings, the literature review identifies the need for development of accessible and relevant educational material; improved communication of disease-specific information between patients and providers, as well as providers and community resources; and strategies to improve the convenience and cost of monitoring devices. However, basic diabetes education (including comprehensive programmes and training to enhance personal understanding of the disease and its implications and advance the practice of self-management) should be regarded as having broad patient-based positive outcomes, but should not be expected to have lasting benefits on glycaemic control [25]. In general, the literature indicates reservations in effects of different measures. For example, exercise consultation is more effective in stimulating exercise behaviour change in the short term than a standard exercise leaflet [71]. Educational interventions should have multiple components. They should aim to improve patients’ sense of self-efficacy and empowerment [72], and develop attitudes towards diabetes that will support the lifestyle changes needed for successful self-management [73]. Interestingly, locally developed education programmes seem to have less effect than interventions developed for the purposes of research studies. This is most likely attributed to the fact that study interventions are applied in a controlled environment, strictly following guidelines and not subject to the limitations of routine practice, but long term impact should also be taken into account [58].

It has become explicit in this review that self-management of diabetes mellitus is physically, intellectually, emotionally and socially demanding. Thus, it will be expected for some patients not to engage with self-management due to personal resources (e.g. health literacy, resilience) or overwhelming personal, family and social circumstances.

### Socioeconomic factors, economic crisis and their impact on self-management

Self-management of type 2 diabetes is referred to in the literature as being strongly interrelated with contextual factors [74]. Research indicates that immigrants might perceive the causes of type 2 diabetes differently compared to people of European origin. An English study [75] found that patients of European origin tend to blame themselves for developing diabetes. In contrast, patients with Pakistani and Indian backgrounds have more varied accounts to explain their diabetes onset, emerging from a more fatalistic sentiment explained as ‘God’s will’ or genetic tendencies, or as “a thread in the fabric of life” often with a strong focus on external stress factors. An opposite perspective to self-blame is seen in accounts from white diabetes patients who externalize the responsibility for the disease. These are primarily cultural issues, but tend to be provoked by economic influences and most notably, poverty [76]. Thus, they are key issues quite visible and of relevance to the capacity to self manage in countries that have been affected by the economic crisis, including Greece, Spain and Bulgaria [77].

Socioeconomic factors impact on the experience of tension, stress and moral dilemmas in patients struggling with their self-management experience in their encounter with health professionals, even to the extent that health care workers in some cases are perceived as adding to the burden of living with a chronic disease [78]. Whilst, the literature identified few studies explicitly seeking data to illuminate the experiences of patients belonging to either socially or economically deprived groups (the concept of deprivation in this review was considered following the WHO guidelines for Europe [79, 80]) both patients and health professionals indicate that lack of social, personal and economical resources are tightly interconnected and affect patients’ self-management. In cases where there is a large social distance between patient and GP’s and other health care workers this might hinder the health professional’s capacity to fully engage with patients’ problems [78]. Recent evidence suggests that lower social class and presumptions of being able to participate are also implicated in chronic conditions overall though this has not been looked at in relation to type 2 Diabetes [81].

These socioeconomic facts might impact further on the tensions and moral dilemmas that patients experience in their efforts to self-manage, and thus add to the burden of having a chronic disease. The literature illuminates patients’ vast experience of the magnitude of economic strain experienced when living with chronic illnesses. There are certain reports from Greece where the impact of economic crisis on the quality and provision of primary care and health care services is high [82]. The economic burden of living with a chronic illness is described by patients as considerable. It affects not only ability to afford essential treatment and medication but also the ability to maintain a healthy lifestyle and quality of life. This burden does not seem to be fully acknowledged by health professionals nor by health politicians. Descriptions of economic burdens will be even more seriously impacted by the global economic downturn.

### Structures and resources in regards to diabetes self-management approaches

Not unexpectedly, there is a variation of health care systems across the participating countries, different funding models and payment systems with General Practitioners (GP’s) as gate keepers in some of them (England, The Netherlands, Bulgaria, Norway), and various governance schemes (primary care trusts in UK, NHS in Bulgaria and Spain, Municipalities in Norway); all seem to have an impact on the management of long term conditions.

Table 3 provides the background for the governance of self-care programmes in the participating countries, as well as the resources that have been allocated to this section. Political commitment and government initiatives have supported many of these approaches. A few of them have been designed for nationwide implementation with regional organisation.

However, certain barriers to change, most probably originating from the health care system structure, seem to exist in a number of countries (vaguely illustrated in the countries that yielded the fewest results in this review: Greece, Bulgaria, Spain and Norway) when discussing how governance and health system organisation promote or inhibit the provision of chronic illness self-management. It should be expected that national health care systems, especially at times when limited resources are available, operate targeted to cost-cutting and identification of effective alternatives.

Within this context, out of the pool of the six participating countries, the majority of research comes from The United Kingdom. The emphasis of care in the UK has now changed from being predominantly reactive to a more preventive management approach, aiming to reduce emergency hospital admissions and encourage patients to make healthier choices about diet, physical activity and lifestyle through self-management of their condition [2]. Despite the long-accepted importance of patient education in diabetes mellitus, an authoritative UK report in 2000 exposed severe deficits in patient education services, describing them as incomplete and not based on current research evidence [83]. As such, self-management interventions have been recognized as a key part of care in the National Service Framework (NSF) in the UK since 2002, spanning the decade studied in this report [84]. Until recently, however, few centres in the UK have offered self-management programmes and rarely were these evidence-based or replicable [29]. In spite of diabetes education offered to all newly diagnosed patients, no universal programme is used. However, the Diabetes Education and Self Management for Ongoing and Newly diagnosed (DESMOND) and Dose Adjustment for Normal Eating (DAFNE) programmes are fairly widely available [25, 85].

In The Netherlands, many Internet-based programmes have been designed, since the most frequently reported self-tests are those for diabetes [86]. In Norway, research on the use of technology, in order to enhance patients’ experience, has been undertaken [87, 88]. Further, there are programmes for patient education located in hospitals [89]. Even though it seems that usage of IT is also the trend in Spain and Greece [60, 68], a literature review has suggested that, despite its potential effect, the use of technology to enhance diabetes self-management is still early in its evolution [90]. The Eurozone economic crisis comes to pose further barriers in the countries that face these limitations, as studies have highlighted the impact of low income status on an effective diabetes self-management [91].

In Bulgaria, a Diabetes Education Program was designed in 1997 to introduce a large-scale unified structured educational program for insulin-treated diabetic patients. Fifty-six in-patient training centers for people with diabetes were established which are still active in all larger cities of the country, while within the required visits to an endocrinologist (which is twice yearly mandatory for patients with diabetes) 25 minutes are devoted to patient education in self-management, under the National Regulation and Instruction [7]. Informational and screening programmes for high-risk population groups are also organized for people with diabetes in Bulgaria [92].

### Organisational structure

Since 2008, there has been an ongoing, global economic recession and a changing European shift towards a demand for cost-effective health policy. It has been an objective to explore the potential impact on the nature and direction of change in health care systems, particularly in relation to chronic illness management. Within this perspective, UK consensus guidelines recommend limited use of self-monitoring of blood glucose (SMBG) in patients with type 2 diabetes using diet and exercise, metformin and/or a glitazone. The study estimates that the potential savings of up to 17 million pound sterling could be made each year if guidelines were followed more closely. There is a need for further research into SMBG use in patients with type 2 diabetes [93]. Moreover, despite effective self-management of diabetes requiring considerable behavioural change and continuous support from health professionals which can be expensive, information technology has the potential to offer cost-effective patient support, but Internet use mostly relies on the active seeking of information [94]. Several implemented initiatives have proven to be cost-effective in their initial operation [66]. However, programmes using the cognitive behavioural approach may be relatively costly when delivered by specialised nurses who have to be trained by behavioural therapists [54].

Expanding on the above concepts, an apparent trend is the delivery of group self-management education. These group-based programmes, delivered in multiple sessions, can assist in the achievement of meaningful changes. Undeniably, as the aforementioned evidence mainly from The Netherlands and the UK indicate, both governmental and various organisations’ initiatives target modern IT services and web-based applications [28, 29, 51, 54]. IT is a way forward to achieve long-term continuous long term self-management support and this trend should definitely be seriously taken into account for the empowerment of new generations of patients, while exploring how to promote community participatory medicine by public institutions and build social capital engaging inter-organisational interactions.

### Shifting sectors and involved professionals

The literature review clearly illustrates a shift in the management of chronic diseases from secondary to primary care that poses stakeholders with new challenges, while the fundamental role of primary care in the management of chronic diseases has been focused on policymakers and politicians in the European Union.

Structural, organisational and socio-economic influences are relevant both to the capacity of patients to self manage and policy and governments and other agencies to support progress in this field. The identification of the importance of socio-economic influences suggest that the gains to date and further progress are likely to be hard to maintain in countries impacted upon most by economic austerity and high levels of poverty and low incomes. Revision of professional roles in multi-disciplinary clinical teamwork in primary care is advocated by European governments. Nurses, GPs, dieticians, psychologists, market analysts and technology experts should collaborate to design or implement specific self-management interventions [12, 45, 56]. However, patients still express fragmentation and lack of coordination and often receive inconsistent and contradictory advice [95]. Certainly, not only patients, but also health care workers in primary care describe lack of communication and collaboration between healthcare professionals. Health professionals relate this lack of cooperation to general challenges of multidisciplinary work, lack of time and burden of administrative work [96]. Both GPs and nurses with a specialist education (diabetic nurse) are described as having a key role in the management of patient with long term conditions, but patients as well as professionals describe issues in inter-professional co-operations between them [96, 97].

Practice nurses play an important role in the nurse-led, shared care which aims to encourage chronically ill people to participate actively in the management of their disease in selecting the organisations and interventions for care [98]. In addition, those that are trained as Diabetes Specialist Nurses not only teach and advise people with diabetes, but also modify and monitor self-management strategies and help develop shared goals, action plans, and skills [99]. Indicatively, in the UK and The Netherlands nurses have enhanced roles and some are trained in empowerment, counselling and communication skills [28, 98], in Greece, it is known that nursing staff in public health care operate within a restricted and task-oriented framework and their educational preparation has little effect in practice role variations and professional needs and this may also be the case for other countries [100]. In the cultural context of these countries, where family coherence is important and can become the thorax to resist challenging situations, family caregivers can play a significant role in sharing and supporting the self-management activities of people with Type 2 diabetes [99].

GP’s should perhaps take responsibility for coordinating these initiatives, reviewing guidelines and safeguarding participation [14, 55]. In addition, findings suggest that although GP’s express the view that they value the increasing patient involvement and the focus on self-management, it is not always prioritized as it clashes with time constraint [101]. GP’s have been found to report that their frustration with patients’ non adherence has resorted to them implementing tactics of “shocking” their patients by using threats as they feel they have no other therapeutic options with which to engage patients [102].

### Usual care pathways

In order for the much desirable behavioural change to be achieved, consistent and long-term education and constant access to relevant information is needed [103, 104]. For example, recent meta-analysis identified that behavioural interventions increased free-living exercise and produced clinically significant improvements in long-term glucose control [105]. These features can be better sustained in the setting of primary care.

Interestingly, over half of the studied initiatives (52%, N = 11/21) have been designed to operate within a primary care framework. Genuine and enduring change requires a multilevel approach, ideally integrated into routine delivery within primary care [12]. Thus, health care services are needed that are better aligned to patient practices of self-management. The above, not unexpectedly, raises the issue of the health care professional-patient relationship and underlines that both university education and continuous medical education can be enhanced with this concept. Various points within the articles included in this review highlight the influence of the setting (especially primary care) as a key determinant in the adoption of self-management behaviours. As the international discussion on health care continues, especially intertwining among the herein participating countries [106–109], each government, along with local organisations, can both learn and extrapolate meaningful lessons to this end. This interaction between health care professionals and patients renders a proper undergraduate education that is not the case for all the European countries.

### Behavioural support

In general, the review revealed that it is still unknown how the gender and education of patients interrelate and affect self-management and the encounter with health care practitioners. In addition, individuals’ perception of social support has received less attention from researchers. In other words; there is a lack of research adapting what might be described as a biopsychosocial approach [110], or a contextual approach [76] or a partnership approach [111] to explore the way people cope with conflicting demands and economic hardship in their striving to achieve balance between managing chronic illness and living a normal life. For example, patients with long term conditions all describe their immediate and extended family as sources of not only emotional, but also practical support [112]. Family support is especially important [113], as the need for practical support related to everyday challenges is described as frequently being ignored by healthcare practitioners [114].

Within the six participating countries, there is evidence that certain actions either as laws, initiatives or programmes have been undertaken. Thus, different management programmes serve actually the same end; specific support for patients with DM in the UK (with Diabetes Education, self-monitoring for Ongoing and Newly Diagnosed, and Dose Adjustment for Normal Eating), the National Framework and contract for patients with DM in Bulgaria, and the telemedicine and technology communication in Norway, The Netherlands and Spain.

### Limitations

Intensive efforts were made to elaborate on the different aspects explored in the review that primarily conveys messages to the countries that have a benefit from this FP7 project. Nevertheless, limitations of this paper should be declared. Realist reviews embrace complexity and the amount of information is sometimes hard to follow. We have attempted to make the extrapolated information more explicit by presenting the findings in different contexts in the Results and Discussion sections. Moreover, a realist review (that incorporates judgment in the interpretation and presentation) is not necessarily standardisable or reproducible in the same sense as a conventional Cochrane review [11]. Of note, while aiming at producing meaningful results at a multinational level, limited availability of studies from certain countries led to the overrepresentation of results from others, especially, The United Kingdom and The Netherlands. Based on the strict systematic method and quality appraisal of included articles, every effort was made not to omit published papers as well as to eliminate bias. Finally, recognising that social interventions are complex and that reproduction of their directions may be difficult is important. Therefore, a meaningful objective is to gather experience and identify what types of interventions work for what kinds of subjects and in which situations.

## Conclusion

The governance literature review, whose results derive from a total of 56 published papers, at least 5,500 individuals residing in the six participating countries, indicates a shift towards patient-centred self-care of diabetes. They identify the need for: development of accessible and relevant education material; improved communication of disease-specific information between patients and providers, as well as providers and community resources; strategies to improve the convenience and cost of monitoring devices; cost-effective designing; and multidisciplinarity in the health care professionals’ approach. Certainly, this work involves multitasking that requires assignment either to national governance bodies and health policy makers or to people accountable for the university training or, lastly, to health care providers themselves.

As demonstrated in this study, a realist review learns from real-world phenomena such as diversity, change, idiosyncrasy, adaptation and programme failure, ad it only leads to tentative recommendations [11]. Thus, this article should be interpreted within the wider context of the whole system approach regarding self-care support and chronic illness management and within the framework of this FP7 project. It is to be complemented with evidence on broader welfare systems and economies of partner countries along with current initiatives and community services. However, there are key messages to all participating countries and especially to those countries affected by the economic crisis. These can assist the pilot intervention that could be designed in the framework of this EU project.

## Authors’ original submitted files for images

Below are the links to the authors’ original submitted files for images. Authors’ original file for figure 1
