# Supplementary figures and images for: Diabetes self-management arrangements in Europe: a realist review to facilitate a project implemented in six countries
Source: BMC Health Serv Res. 2014 Oct 2;14:453. doi: 10.1186/1472-6963-14-453 (PMC4283086; doi:10.1186/1472-6963-14-453)

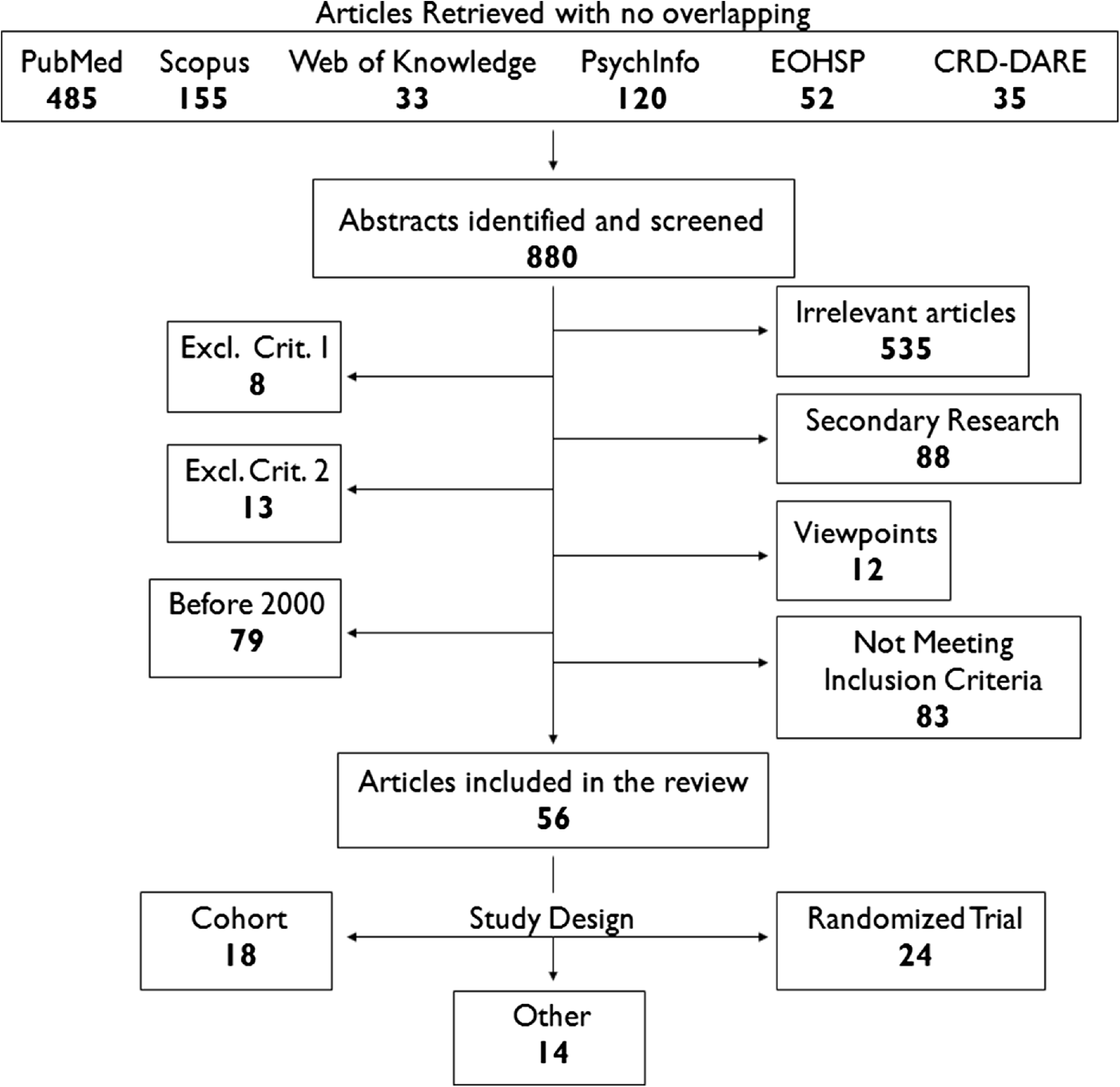

Supplement: Supplementary file 1 — Authors’ original file for figure 1 [file 12913_2014_3550_MOESM1_ESM.tiff]
